# Clinical Presentation, Management and Outcome of Cerebral Echinococcosis in Children: A Systematic Review and Meta-Analysis

**DOI:** 10.3390/pathogens14111144

**Published:** 2025-11-11

**Authors:** Roberta Leonardi, Alessandra Curatolo, Manuela Lo Bianco, Alessia Migliore, Grete Francesca Privitera, Alfredo Pulvirenti, Giuseppe Nunnari, Andrea Marino, Serena Spampinato, Antonino Maniaci, Pasqua Betta, Martino Ruggieri, Agata Polizzi, Piero Pavone

**Affiliations:** 1 Postgraduate Training Program in Pediatrics, University of Catania, 95125 Catania, Italy; alessiamigliore16@gmail.com; 2 Neonatal Intensive Care Unit, AOU Policlinico G. Rodolico San Marco, 95123 Catania, Italy; mlbetta@yahoo.it; 3 Department of Clinical and Experimental Medicine, School of Medicine, University of Catania, 95123 Catania, Italy; alessandracuratolo97@gmail.com; 4 Unit of Pediatric Clinic, Department of Clinical and Experimental Medicine, University of Catania, 95123 Catania, Italy; lobiancomanuela@gmail.com (M.L.B.); m.ruggieri@unict.it (M.R.); agata.polizzi1@unict.it (A.P.); 5 Bioinformatics Unit, Department of Clinical and Experimental Medicine, University of Catania, 95123 Catania, Italy; grete.privitera@unict.it (G.F.P.); alfredo.pulvirenti@unict.it (A.P.); 6 Unit of Infectious Disease, Department of Clinical and Experimental Medicine, AOU Garibaldi, University of Catania, 95123 Catania, Italy; giuseppe.nunnari1@unict.it (G.N.); andrea.marino@unict.it (A.M.); serenaspampinato93@gmail.com (S.S.); 7 Department of Medicine and Surgery, University of Enna Kore, 94100 Enna, Italy; tnmaniaci29@gmail.com

**Keywords:** echinococcosis, cerebral echinococcosis, hydatic cysts, pediatric neuroparassitosis, pediatric echinococcosis

## Abstract

Background: Cerebral echinococcosis is a rare, potentially serious parasitic disease in children, that can lead to intracranial hypertension, focal neurological deficits, seizures, and severe complications. We conducted a systematic review and meta-analysis on diagnostic, therapeutic approaches, and outcomes in pediatric cerebral echinococcosis. Methods: A systematic search was performed on PubMed, Scopus, and Web of Science, selecting English studies on children (0–18 years). Studies describing clinical, imaging, surgical, pharmacological, and outcome data were eligible. Statistical analyses (Fisher’s exact and chi-square tests) were performed in R. Results: A total of 100 studies with 462 pediatric patients met the inclusion criteria. High-resolution imaging has largely replaced invasive diagnostics; MRI-based diagnosis correlated with better outcomes. Headaches, vomiting, papilledema, seizures, and hemiparesis were common. Surgical cysts’ removal remained the main therapy. Additional treatment with albendazole was associated with a higher probability of good outcome (*p* < 0.001). A greater number of cyst localizations was significantly associated with a worse prognosis (*p* < 0.001). Overall mortality was 8.9%, while approximately 2/3 of patients achieved a good outcome. Conclusions: Advances in non-invasive imaging, refinement of surgical technique, and targeted antiparasitic therapy improved outcomes. Nevertheless, heterogeneous reporting and the prevailing paucity of evidence limit definitive recommendations. Prospective multicenter studies are needed to refine treatment and develop pediatric-specific guidelines.

## 1. Introduction

Echinococcosis is a zoonotic parasitic infection caused by tapeworms of the genus Echinococcus. According to the World Health Organization (WHO, 2021), four morphologically and biologically distinct species are of medical importance in humans: *Echinococcus granulosus*, *E. multilocularis*, *E. oligarthrus*, and *E. vogeli* [1]. The most common species causative agent of human echinococcosis is *E. granulosus* and *E. multilocularis*. The life cycle of Echinococcus involves two types of hosts. Carnivores such as dogs and other canids serve as definitive hosts, harboring the adult tapeworm in their intestines. Herbivorous and omnivorous animals act as intermediate hosts, becoming infected by ingesting parasite eggs excreted in the feces of definitive hosts [1]. In these animals, the larval form develops as a hydatid cyst (cysta hydatigena) within visceral organs, most commonly the liver and lungs. Humans are accidental intermediate hosts, acquiring the infection in the same way as other intermediate hosts, typically through ingestion of contaminated food, water, or soil, or through direct contact with infected dogs. The development of larval cysts in human tissues may take years before clinical symptoms appear [1,2,3]. Among the most affected individuals are adults with immunosuppression due to organ transplantation and consequent immunosuppressive therapy, individuals with HIV infection, and children with more sensitive immune system [3,4]. This parasite is found in regions of the world where close interactions between humans, livestock, and dogs facilitate its spread, such as in South America, Australia, India, China, East Africa, and the Mediterranean Basin [2]. The infection is caused by tapeworms found in cats, foxes, and particularly in domestic dogs, which are the definitive hosts, while herbivores and rodents act as intermediate hosts. Humans are accidentally infected by ingesting food or water contaminated with parasite eggs [5]. Once ingested, the eggs release oncospheres that penetrate the intestinal mucosa and travel through the portal circulation, eventually localizing in the liver in 75% of cases. In 15% of cases, the parasite reaches the lungs and, in rare cases, can spread to the central nervous system [6]. Although the liver and lungs are the most commonly affected organs, rare sites such as the central nervous system (CNS) may also be involved; in fact, cerebral echinococcosis has an incidence of 0.5–3% of all cases [7]. In the brain, the parasite forms hydatid cysts that grow slowly, without triggering a marked inflammatory response, making this condition different from neurocysticercosis, where a much stronger immune reaction occurs [3]. These intracranial lesions account for approximately 2% of all space-occupying lesions in countries with a higher incidence of echinococcosis [7]. In 1% of cases, the localization is instead in the spine [8]. Cerebral cysts are generally large, well defined, and singular, without significant perilesional edema. The growth of the cyst in the brain is generally around 1 cm per year, although significantly faster growth has been reported [9,10]. In some rare cases, spontaneously degenerating hydatid cysts have been documented, leading to a reduction in cyst size [9]. Clinical symptoms depend on the size, number, and location of the cyst, and may include signs of intracranial hypertension such as headache, nausea, vomiting, papilledema, seizures, and focal neurological deficits [11]. Cyst rupture can lead to severe allergic reactions or anaphylaxis. In more developed countries, diagnosis can be particularly complex due to its near-total eradication. However, there has been a recent increase in its incidence as a consequence of rising migration flows. Early identification and treatment are crucial to prevent complications and the progression of the parasitic infection [7]. Diagnosis is primarily based on neuroimaging, which reveals large, well-defined, fluid-filled cysts, while serological tests, although useful, may not be effective in cases of exclusive cerebral involvement [8]. The main treatment consists of surgical removal of the cysts, and the use of drugs such as albendazole, praziquantel, or mebendazole [9]. The drug treatments not only help to reduce the size of the cysts but can also prevent potentially dangerous allergic reactions in the case of rupture of the cysts [7]. Therefore, a systematic review focusing on pediatric cerebral echinococcosis is needed to clarify the clinical presentations and diagnostic challenges in children, to explore the most effective therapeutic approaches and adjunctive treatments, and to examine the relationship between diagnostic methods, treatment, and prognosis. By synthesizing the existing literature, this review aims to provide a comprehensive understanding of epidemiology, optimal management, and prognostic factors related to pediatric cerebral echinococcosis, ultimately guiding clinicians and researchers toward evidence-based best practices. This study analyzes the effectiveness of new therapeutic protocols for this disorder through a systematic review and meta-analysis. The main objective is to examine diagnostic techniques, therapeutic strategies, and clinical outcomes, with the aim of optimizing treatments and promoting early diagnosis.

Objectives:

- To evaluate the clinical presentations, diagnostic approaches, and treatment strategies used in pediatric patients (0–18 years) with cerebral echinococcosis.
- To analyze post-treatment outcomes including treatment-related complications.
- To identify prognostic factors, such as cyst burden/location, treatment timing, the correlation between specific treatments and outcomes and between instrumental diagnosis and outcomes.

## 2. Materials and Methods

### 2.1. Systematic Review and Meta-Analysis

We carried out a systematic review and meta-analysis focused on cerebral echinococcosis following the guidelines outlined in the Preferred Reporting Items for Systematic Reviews and Meta-Analyses (PRISMA) [12,13].

### 2.2. Search Strategy

**Figure 1:**
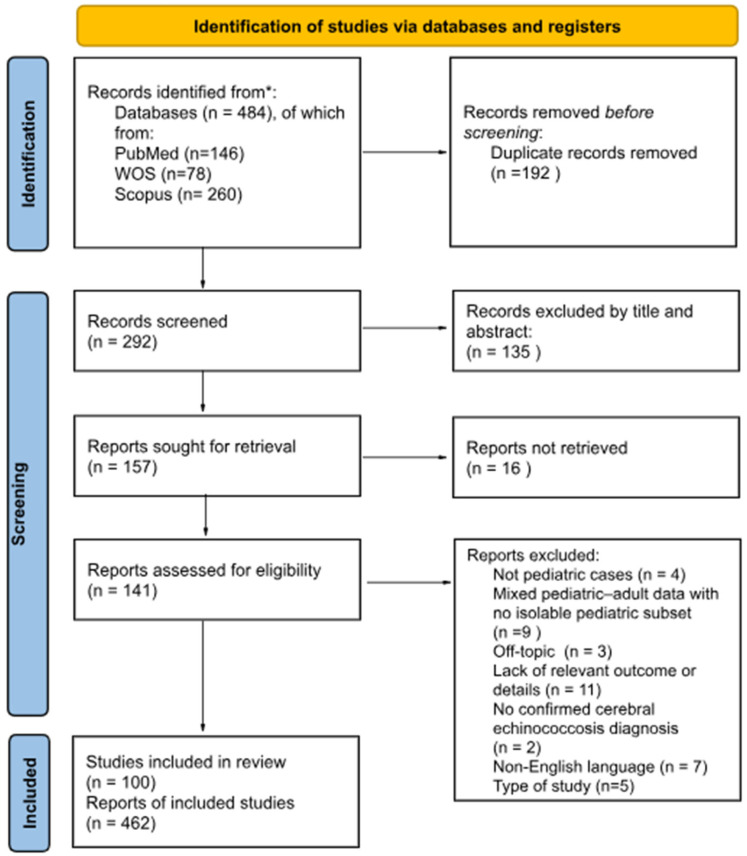
PRISMA 2020 Flow Diagram from * Page MJ, McKenzie JE, Bossuyt PM, Boutron I, Hoffmann TC, Mulrow CD et al. The PRISMA 2020 statement: an updated guideline for reporting systematic reviews.

An exhaustive literature search was performed in three databases by (PubMed/MEDLINE, Scopus, and Web of Science) by two independent revisors (RL, AC) until 12 March 2025, including all terms possibly linked to echinococcal infections and central nervous system involvement in children. In particular, we utilized the following search string: (“echinococcosis” OR “echinococcus”) AND (“nervous system” OR “cerebral”) AND (pediatric OR child). No time filters were applied, but we used a language filter for English, a species filter for “Humans”, and an age filter for “0–18 years”, where database functionalities permitted. Additionally, the gray literature, including reference lists from relevant articles and conference proceedings, was reviewed to ensure completeness. To refine the search results, we scanned article titles, abstracts, and indexing terms. All records were managed with Zotero 7, where duplicates were removed through manual method. The stepwise selection of studies, following the PRISMA recommendations, is illustrated in Figure 1.

### 2.3. Eligibility Criteria

1. Population
  - Patients in pediatric age (0–18 years) with echinococcal infection and neurological involvement
2. Intervention/Exposure
  - Research documenting natural history, diagnostic techniques, treatment procedures, or clinical outcomes in echinococcosis with CNS or intracranial manifestations.
3. Comparison
  - Inclusion of studies without comparator groups was permitted, reflecting the variability in clinical practice and the overall rarity of cerebral echinococcosis.
4. Outcomes
  - Neurological features (e.g., location of cysts, elevated intracranial pressure, seizures, focal deficits).
  - Treatment outcomes (e.g., cyst regression or resolution, recurrence, cyst’s complications).
  - Other clinically relevant measures, such as mortality or outcome.
5. Study Design
  - Eligible designs encompassed case reports, case series, cohort analyses, case–control studies, and interventional trials (both randomized and non-randomized) presenting outcome data.
  - Systematic reviews of primary studies were considered if they contributed unique, original insights or unpublished findings.
6. Language
  - Only studies published in English or those with sufficiently detailed English summaries were included.

### 2.4. Study Selection

Study selection was performed independently by two reviewers (RL, AC); any discrepancy between the two was resolved by a third reviewer (MLB). Initially, the screening of all articles was performed by titles and abstracts. Any article that clearly did not meet the inclusion criteria or was outside the scope of echinococcal infections of the CNS was discarded. When there was uncertainty about a particular publication, it was retained for full-text evaluation. Full-text copies of all potentially relevant papers were then reviewed in detail by the same group of reviewers, who applied the previously established selection criteria. Reasons for excluding any articles during this phase were recorded; in particular, they were as follows: the clinical case was not pediatric; the full text was unavailable; the study involved mixed pediatric and adult populations with no possibility of isolating pediatric data; the study focused on different parasites or did not address neurological involvement, therapy, or outcomes; the article lacked original data (e.g., letters, purely radiological reviews); the report was not in English; or there was no confirmed diagnosis of cerebral echinococcosis.

### 2.5. Data Extraction

A standardized, electronic form was used for the data extraction, in order to collect the following data: patient baseline characteristics, study design, sample size, patient demographics, neurological symptoms, instrumental diagnosis, medications (e.g., albendazole), surgical management, adjunctive treatments (e.g., steroids), cyst resolution rate, recurrence, neurological complications, survival data. Inconsistencies or missing details (such as specific treatment durations or incomplete demographic data) were noted, and any disagreements in data extraction were resolved through group consensus. A detailed PRISMA checklist is included in the Supplementary Material, ensuring adherence to the key requirements for transparent reporting.

### 2.6. Statistical Analysis

A qualitative synthesis of all included studies was performed, describing the range of clinical neurological manifestation, interventions, and outcomes. Where more studies reported comparable outcome measures in a sufficiently homogeneous manner, we conducted a meta-analysis. Statistical descriptive analysis was performed including mean, median, SD, IQR, relative, and percentage frequencies. Sample comparisons have been executed using R (v4.0.0) through Fisher-exact test and Chi Square-test.

### 2.7. PROSPERO Registration

Since our analysis was restricted to data from previously published research, separate ethical approval was unnecessary. This systematic review was submitted for registration on the International Prospective Register of Systematic Reviews (PROSPERO): registration number 1009491. The protocol and any related materials for this review are available upon request from the corresponding author.

## 3. Results

### 3.1. Systematic Review and Characteristics of Included Studies

A total of 484 records were identified through database searching. After manually removing duplicates (*n* = 192), 292 records remained. Based on a review of titles and abstracts, 135 of these were excluded, leaving 157 studies for more detailed assessment. Of these, 16 records could not be retrieved; then, 141 articles were assessed for eligibility. Following the full-text review, 41 articles were excluded (as detailed in the PRISMA flow diagram), for reasons such as not focusing on pediatric patients, lacking essential outcome data, being off-topic, or not meeting the language or diagnostic criteria required by our protocol. The reasons of exclusions were as follows: mixed pediatric–adult data with no possibility to isolate pediatric outcomes, not pediatric cases (*n* = 13); no original and relevant data, off-topic, type of study (e.g., letter, editorial, or narrative review,) (*n* = 19); non-English language (*n* = 7); no confirmed diagnosis of cerebral echinococcosis (*n* = 2). Consequently, 100 studies met all the inclusion criteria, encompassing data on 462 pediatric patients with echinococcosis. These articles form the basis of the subsequent analyses [7,9,14,15,16,17,18,19,20,21,22,23,24,25,26,27,28,29,30,31,32,33,34,35,36,37,38,39,40,41,42,43,44,45,46,47,48,49,50,51,52,53,54,55,56,57,58,59,60,61,62,63,64,65,66,67,68,69,70,71,72,73,74,75,76,77,78,79,80,81,82,83,84,85,86,87,88,89,90,91,92,93,94,95,96,97,98,99,100,101,102,103,104,105,106,107,108,109,110]. When it comes to the type of studies, the following were included: 8 observational/retrospective studies, 25 case series, and 67 case reports. Among the 100 included articles, 8 (8.0%) provided Level 3 evidence, according to the classification of the Oxford Center for Evidence-Based Medicine (2011), while the remaining 92 (92.0%) were classified as Level 4 evidence. This categorization reflects the predominance of case reports, case series, or observational designs with relatively lower methodological rigor. The date range of publications was 1965–2025.

### 3.2. Population Characteristics

Of the 100 included studies, only 22 specified the duration of the study; among these, the duration ranged from 3 to 44 years, with a mean study duration of 15.6 ± 11.5 years, and a median of 14.0 years. Of the 100 included studies, 68 did not specify ethnicity, but in 32 the following was described: Iranian was the most frequently reported (*n* = 4), followed by African (*n* = 3) and South African (*n* = 3); Albanian, Asian, Chinese, and Indian each appeared in 2 studies. In the 14 remaining studies, ethnicity was Afghan, Arab, Argentine, Asian (Turkmenistan), British, European, Iraqi, Italian, North African, Romanian, Spanish, Syrian, Syrian/Turkish, and Turkish. About the age at diagnosis, it was reported only in 193 patients (41.8%). Among these 193 patients, ages ranged from 2 to 18 years, with a mean ± SD of 9.4 ± 3.8 years and a median (IQR) of 9 (7–12) years. For ease of interpretation, we grouped these 193 values into the following four categories: <5 years: 15 patients (7.8%); 5 to <10 years: 92 patients (47.7%); 10 to <15 years: 61 patients (31.6%); 15 to <20 years: 25 patients (13.0%). Notably, over half (55.5%) of patients with available data were diagnosed before reaching 10 years of age. Further, sex distribution analysis showed a slight prevalence of males: 199 patients were female (43%), 263 were male (56%). Data on the Echinococcus species was available for 159 cases; of these, *Echinococcus granulosus* was identified in 74 patients (46.5%), while *E. multilocularis* was reported in 63 (39.6%).

### 3.3. Clinical Presentation

**Figure 2:**
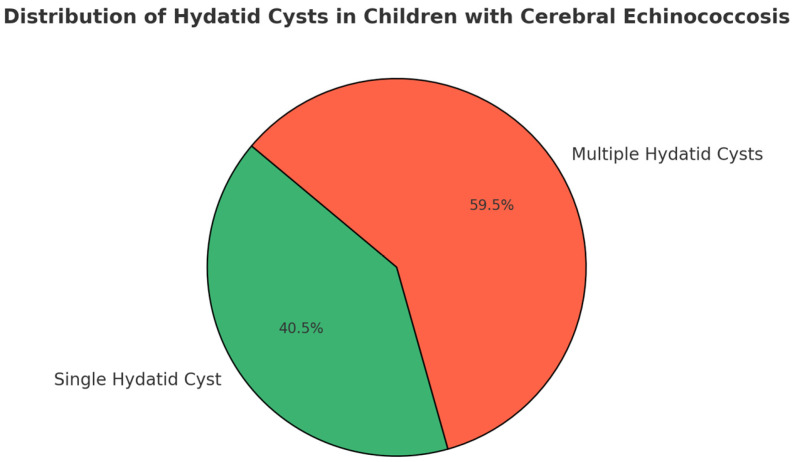
Distribution of hydatid cysts in children with cerebral Echinococcosis.

Regarding the clinical diagnosis, 187 children (40.5%) were diagnosed with a single hydatid cyst, while 275 (59.5%) with multiple hydatid cysts (Figure 2).

**Figure 3:**
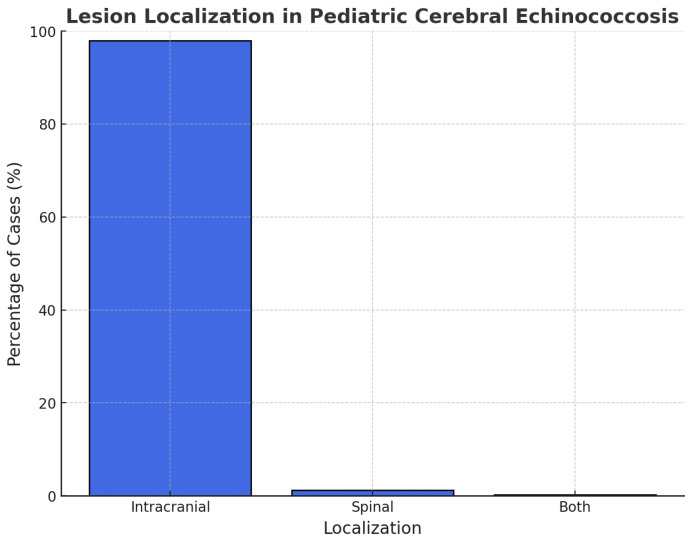
Lesion localization in cerebral Echinococcosis in pediatric patients.

We evaluated the localization of the cyst and divided them into intracranial and spinal groups. The localization was intracranial in 98% of cases and spinal in 1,2%, while only one patient presented both (0.2%) (Figure 3). It was not possible to retrieve data on precise anatomical localization in 295/462 patients. Among the cases reporting precise anatomical localization, the parietal region was most commonly involved (36%), followed by frontal (23%), temporal (23%), and occipital (9%). The cerebellar region was affected in 7 cases (1.5%), the ventricles in 12 (2.6%), the parasellar area in 3 (0.6%), and posterior cranial fossa cysts in 2 (0.4%). Less frequent sites included the brainstem, septum pellucidum, and thalamus (1 case each). Dorsal lesions were noted in 4 patients (0.8%), lumbar involvement accounted for 2 cases (0.4%), while cervical localizations were not reported in this dataset (0%). Additionally, we wondered how many patients presented two or more localizations: 144 patients (86%); while 14% were characterized by a single localization.

**Figure 4:**
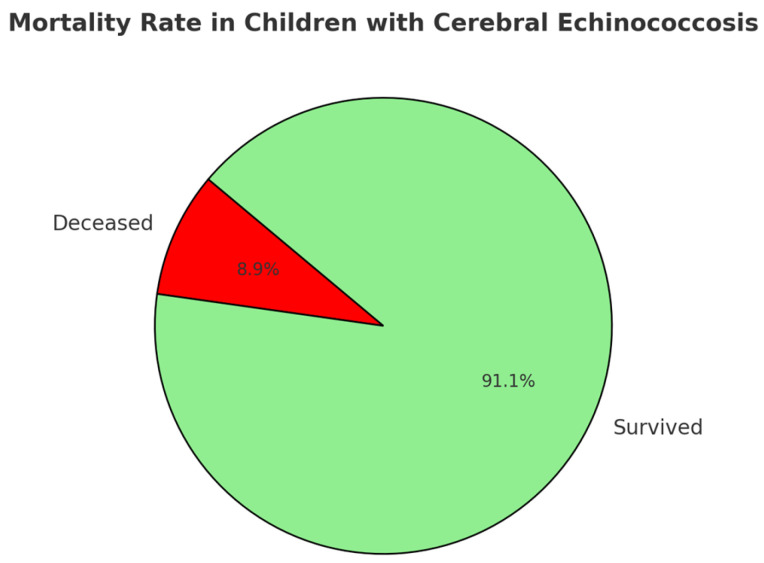
Mortality rate in children with cerebral Echinococcosis.

A visual summary of mortality rate in pediatric cerebral echinococcosis is provided in Figure 4. As shown, a minority of patients (*n* = 41) experienced fatal outcomes, including perioperative deaths in three cases. For those who did not experience perioperative death, the mean time to death following treatment (surgical and/or pharmacological) was 9.94 months (standard deviation, 16.93), with a median of 3 months and an interquartile range (IQR) of 0.23–12 months.

In terms of overall prognosis, we stratified patients into the following two outcome categories: “good outcome,” indicating satisfactory recovery without long-term sequelae, and “bad outcome,” defined by the occurrence of at least one complication (including death). Based on these criteria, 292 patients (63.2%) were classified as having a good outcome, whereas 170 patients (36.8%) were considered to have a bad outcome. The most common complications included seizures (1.5%) and hemiparesis (0.9%), with a wide range of additional issues—such as hydrocephalus, meningitis, cranial nerve deficits, visual disturbances (including bilateral blindness), recurrence, and anaphylactic shock—each reported in fewer than 1% of cases.

**Figure 5:**
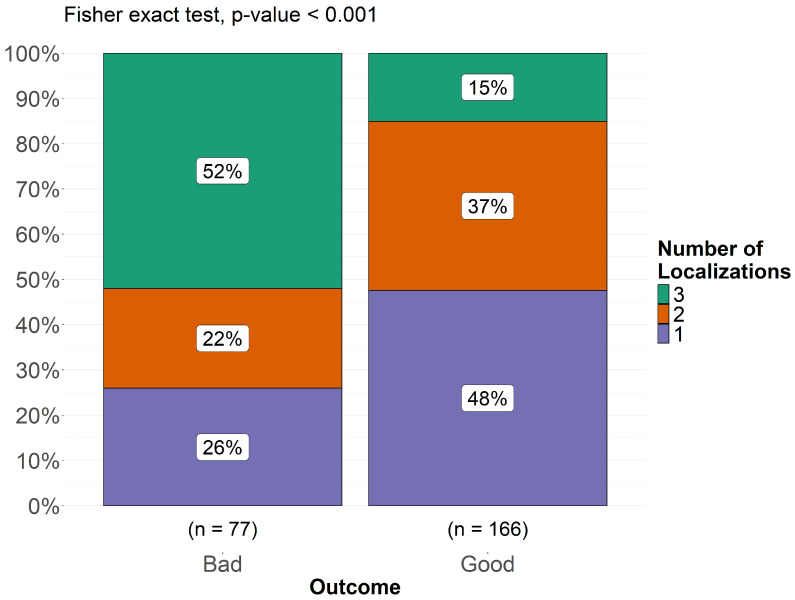
Distribution of cyst extension (single, two, or three localizations) in relation to clinical outcomes.

A chi-square test was performed to assess the association between cyst extension (classified as single, two, or three localizations) and clinical outcomes (good vs. bad outcome), as shown in Figure 5. The analysis yielded a statistically significant result (*p* < 0.001), demonstrating a direct proportionality between greater cyst extension and worsening clinical outcome. Among patients with a bad outcome, 26% had a single localization, 22% had two localizations, and 52% had three localizations. Conversely, in the good outcome group, 48% of patients had a single localization, 37% had two localizations, and only 15% had three localizations. These findings suggest that a higher number of localizations is significantly associated with poorer prognosis in pediatric cerebral echinococcosis. We collected data on the presence or absence of the following neurological signs and symptoms in this patient population: headache, raised intracranial pressure, vomiting, papilledema, hemiparesis, weakness of lower limbs, seizures, ataxia, aphasia, cerebellar signs, pyramidal signs, ptosis, optic trophy, paralysis of cranial nerves, neuropsychiatric symptoms (personality changes/low school performances/intelligence impairment), visual problems, alteration/reduction in consciousness, cranial asymmetry, and hemichorea. For each of these signs/symptoms, we recorded whether it was not described (“N/A”), whether it was described as present, or whether it was described as absent. The relative frequencies and percentages of each sign/symptom were calculated based on the total number of cases in which the sign/symptom was described, excluding the number of patients for whom the sign/symptom was not reported.

As illustrated in Table 1, the most frequent manifestations were headache, raised intracranial pressure, vomiting, and papilloedema, consistent with space-occupying lesions. Motor deficits, such as hemiparesis and lower limb weakness, were also common. A substantial proportion of patients exhibited seizures, cerebellar involvement, cranial nerve palsies, or optic atrophy. Neuropsychiatric abnormalities, including personality changes, reduced school performance, or cognitive impairment, were reported in most described cases. Visual disturbances were also frequent, and nearly one-third of patients presented with altered or reduced consciousness; coma was documented in a few severe cases. In 52 cases (69%), there was an alteration or reduction in the state of consciousness, which did not occur in 23 patients; specifically, 7 children developed coma. Only in 8 children was the Glasgow Coma Scale (GCS) specified; according to this, 3 patients presented a minor brain injury (GCS > 13), while 2 cases had a moderate brain injury (GCS 9–12), and 3 had a GCS comparable to a coma status (GCS 3–8).

### 3.4. Instrumental Diagnosis

**Figure 6:**
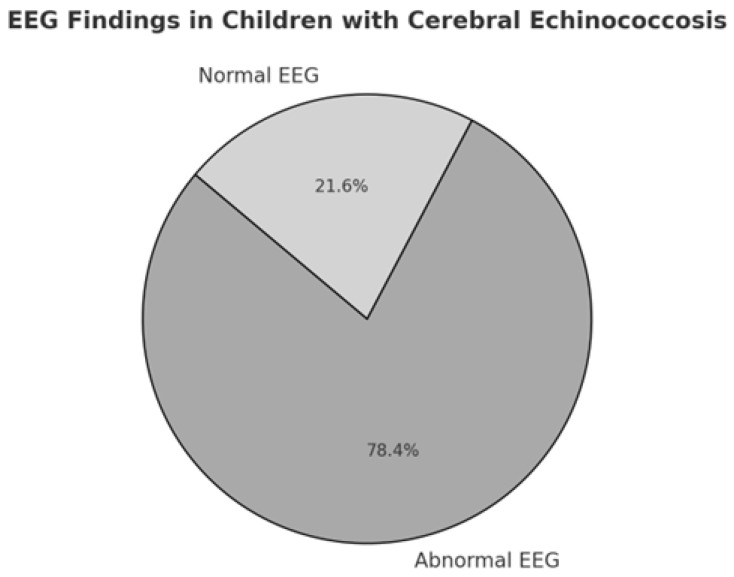
EEG findings in children with cerebral Echinococcosis.

**Figure 7:**
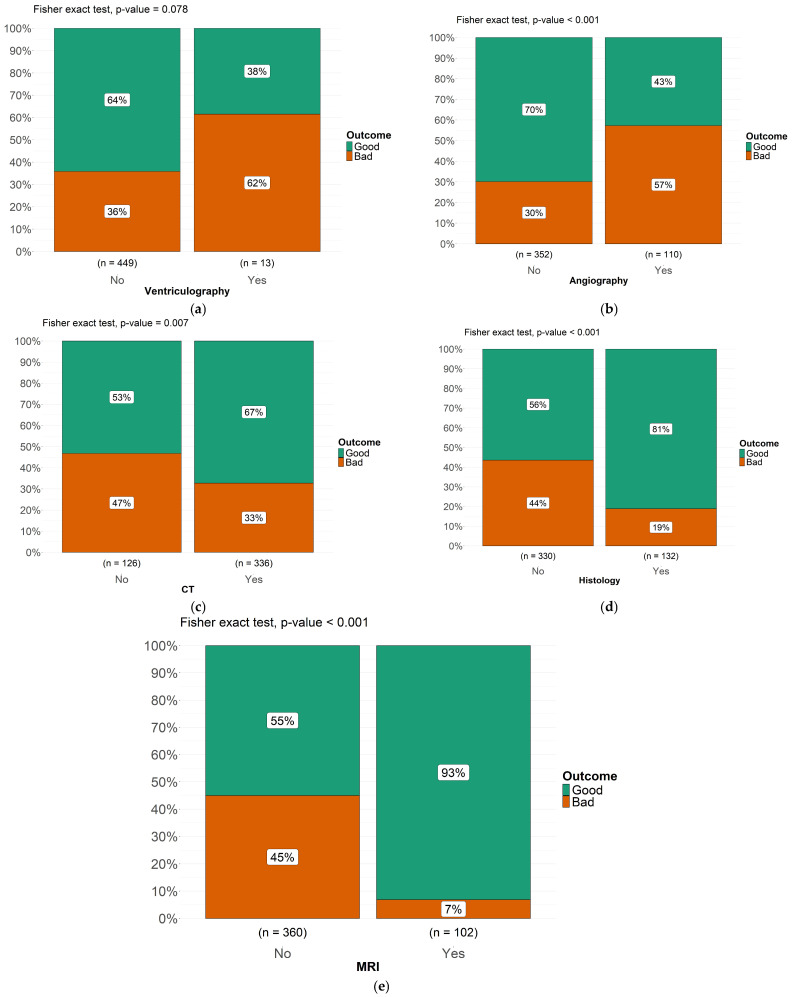
(**a**–**e**) Impact of different diagnostic modalities on clinical outcomes in pediatric cerebral echinococcosis. (**a**) Ventriculography. (**b**) Angiography. (**c**) CT. (**d**) Histology. (**e**) MRI.

When explicitly executed, EEG showed abnormalities in 29 cases, while it was normal in 8 cases (Figure 6). Regarding the diagnosis, initially based on the “Casoni’s test”, it was used in 33 of reported cases and specifically it was positive in 12, while negative in 11 of them. The instrumental diagnosis was previously based on angiography, which was described in 278 cases (particularly positive in 102 of them, 37%) and on ventriculography, described in 210 cases (positive in 13 cases, 6%). Then, the instrumental diagnosis was based on CT in 378 children (positive in 89% of cases), MRI in 220 patients (positive in 44%). Histology has allowed diagnosis in 145 patients, 55% of those in which it was performed. Furthermore, we investigated which type of instrumental diagnostic assessment had the greatest impact on prognosis in pediatric patients with cerebral echinococcosis, based on cases reported in the literature (Figure 7a–e). Furthermore, we evaluated the prognostic impact of the diagnostic methods historically used for cerebral echinococcosis, including ventriculography, which was once employed to visualize ventricular displacement or obstruction caused by intracranial cysts. Based on the cases reported in the literature, we assessed whether the use of ventriculography was associated with worse clinical outcomes. Fisher’s exact test showed the following statistically significant association (*p* = 0.078): among patients who underwent ventriculography as part of the diagnostic workup, 62% experienced a bad outcome, which is potentially due to its invasive nature and related complications. Patients diagnosed via angiography had a bad outcome in 57% of cases (Fisher’s exact test, *p* < 0.001); conversely, computed tomography (CT) diagnosis was associated with a good outcome in 67% of cases (Fisher’s exact test, *p* < 0.007), indicating a statistically significant correlation between CT-based diagnosis and better clinical prognosis. Patients diagnosed through histological examination exhibited a good outcome in 81% of cases (Fisher’s exact test, *p* < 0.001), reinforcing the role of histopathology in guiding effective treatment strategies.

Finally, among patients diagnosed via magnetic resonance imaging (MRI), 93% experienced a good outcome (Fisher’s exact test, *p* < 0.001), highlighting the strong predictive value of MRI in early identifying cases leading to a favorable prognosis. These findings emphasize the importance of selecting non-invasive and high-resolution imaging techniques, such as MRI, to allow early recognition of these conditions and optimize clinical management and improve patient outcomes (Figure 7a–e).

### 3.5. Treatment and Outcome

All the reported patients were surgically treated (*n* = 462), but in 13 cases it was not possible to retrieve the single data regarding the type of surgery. When specified, the type of surgery was characterized by the following: cyst aspiration and wall removal (5%); only asportation through hydrodissection (93%); other (laminectomy, laminoplasty, curettage) (2%).

**Figure 8:**
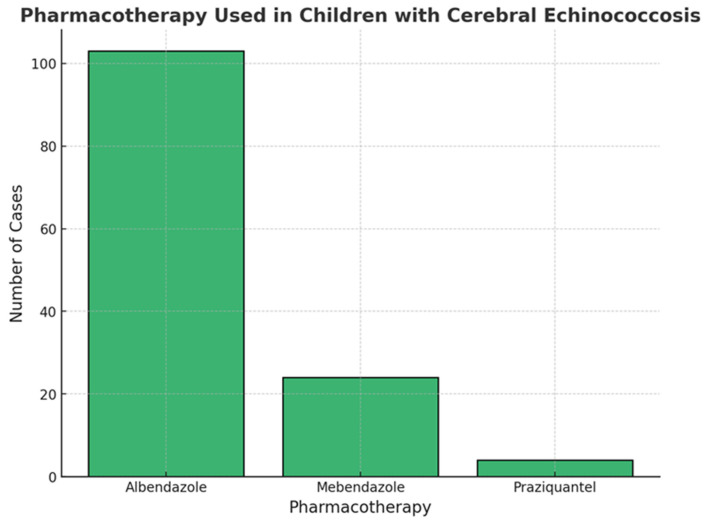
Anti-parasitic drug-therapy used in children with Cerebral Echinococcosis.

As shown in Figure 8, the most used anti-parasitic drug in children affected by cerebral Echinococcosis was albendazole (*n* = 103; 46%), followed by Mebendazole (*n*= 24; 11%) and Praziquantel (*n* = 4; 2%). These patients were also treated with a support pharmacotherapy; in particular, 50 patients were treated with antiepileptic drugs (24%), and 6 cases with corticosteroids (3%).

**Figure 9:**
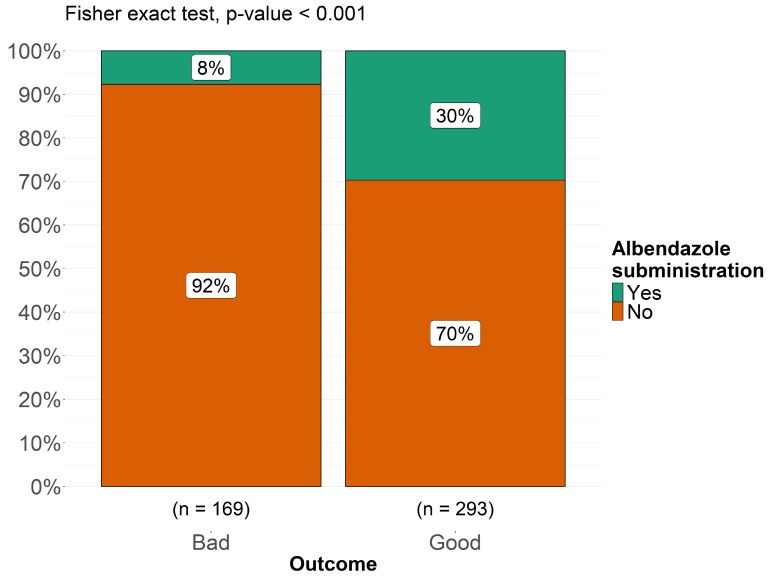
Distribution clinical outcomes (good vs. bad) in relation to Albendazole administration in pediatric patients with cerebral echinococcosis.

**Figure 10:**
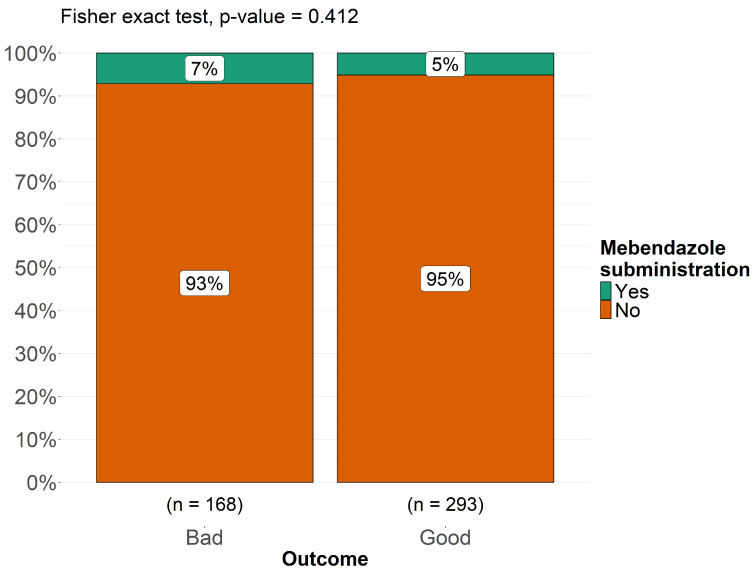
Distribution clinical outcomes (good vs. bad) in relation to Mebendazole administration in pediatric patients with cerebral echinococcosis.

As shown in Figure 9 and Figure 10, albendazole was significantly associated with a better prognosis (*p* < 0.001), while mebendazole showed no significant correlation (*p* = 0.41). Fisher’s exact test was conducted to compare the antiparasitic drugs most commonly used in this pediatric cohort in relation to treatment outcomes, categorized as “good” and “bad.” The analysis revealed that 30% of patients with a good outcome had been treated with albendazole, whereas only 8% of patients who received albendazole experienced a bad outcome (*p* < 0.001), suggesting a statistically significant association between albendazole use and favorable prognosis. Conversely, for mebendazole, the proportion of patients receiving this drug was 5% in the good outcome group and 7% in the bad outcome group (*p* = 0.41), indicating no significant correlation between mebendazole administration and clinical outcome (Figure 10). We investigated whether there was a relationship between individual neurological symptoms and clinical outcomes, classified as “good outcome” and “bad outcome,” to assess whether neurological symptoms influenced the final prognosis and to delineate the neurological phenotype of patients within each outcome category. To conduct this analysis, we applied Fisher’s exact test for each comparison between a single neurological symptom and the two outcome categories. None of these analyses yielded a *p*-value < 0.05, indicating that no significant differences were found between the two outcome groups when comparing individual neurological symptoms. Among patients with a bad outcome, 96% presented with headache, compared to 94% of those with a good outcome. Notably, none of the patients with a bad outcome exhibited cerebellar signs, whereas cerebellar signs were observed in 53% of patients with a good outcome. Papilledema was present in 90% of patients with a bad outcome and 87% of those with a good outcome. Weakness of the lower limbs was observed in 79% of patients with a good outcome, compared to 67% of those with a bad outcome. Aphasia was present in all patients with a bad outcome, while 52% of those with a good outcome did not exhibit aphasia. Ataxia was reported in 74% of patients with a good outcome and in 50% of those with a bad outcome. Seizures were equally present in both groups (75%), with no differences between the two outcome categories. Hemiparesis was present in 90% of patients with a good outcome and 87% of those with a bad outcome. Ptosis was more prevalent in patients with a bad outcome (75%), while it was observed in only 30% of those with a good outcome. Neuropsychiatric symptoms were significantly more frequent in patients with a bad outcome (80%) compared to 36% of those with a good outcome. Altered consciousness was observed in 67% of patients with a bad outcome and in 53% of those with a good outcome, while coma was present in 40% of subjects with a bad outcome and in 20% of those with a good outcome. Visual problems were reported in 94% of patients with a bad outcome, whereas 75% of those with a good outcome experienced similar issues. Optic atrophy was present in 75% of patients with a bad outcome but in only 36% of those with a good outcome. Finally, pyramidal signs were present in 88% of patients with a bad outcome and in 69% of those with a good outcome. These findings suggest that no single neurological symptom is a definitive predictor of clinical outcome in pediatric cerebral echinococcosis. While certain symptoms appeared to be more prevalent in either the “good outcome” or “bad outcome” groups, none of these associations reached statistical significance, indicating that neurological presentation alone may not be sufficient to determine prognosis.

## 4. Discussion

This systematic review and meta-analysis, which consolidates data from 100 studies encompassing 462 children, is the first focused pediatric cerebral echinococcosis, to date. Our findings provide a comprehensive overview of clinical presentation, diagnostic modalities, and therapeutic outcomes in this rare but significant entity.

Across the studies, neurological symptoms emerged as pivotal indicators of disease presence and severity. In the subset of patients where data were reported, headache was the most prevalent symptom (97%), often coupled with raised intracranial pressure (96%), vomiting (96%), and papilledema (95%) with reduced visual acuity and visual blurring. This constellation underscores the role of intracranial hypertension as a frequent clinical manifestation, especially among children with sizable or multiple cysts. In very young children, intracranial hypertension can cause increased skull circumference and cranial asymmetry (84%) due to the pliability of their skulls. Aphasia occurred in 7 children and it was noted in frontal lobe lesions, typically manifesting as Broca’s aphasia. In one case [103], a child with multiple intracranial cysts and an eyelid cyst developed frontal lobe syndrome, characterized by uncontrolled laughter and crying. When explicitly executed, EEG showed abnormalities in 78.4% of cases (*n* = 29), including focal slowing and beta-theta waves, while it was normal in 8 cases. Only in 8 children was the Glasgow Coma Scale (GCS) specified; according to this, 3 patients presented a minor brain injury (GCS > 13), while 2 cases had a moderate brain injury (GCS 9–12), and 3 had a GCS comparable to a coma status (GCS 3–8). Altered consciousness was more frequent in patients with a bad outcome (67% vs. 53%), with coma occurring twice as often in this group (40% vs. 20%). These findings suggest that a greater degree of neurological dysfunction, particularly affecting mental status, correlates with worse clinical trajectories. Similarly, ptosis was observed in 75% of those with a bad outcome, but only in 30% of those with a good outcome, which may reflect more widespread neuroanatomical involvement in severe cases. Additional deficits included hemiparesis (94%), weakness of the lower limbs (88%), and seizures (91%), reflecting both cortical and subcortical involvement. More focal signs—such as ptosis (43%), optic atrophy (83%), and cranial nerve paralysis (79%)—demonstrate the broad neuroanatomical impact of hydatid lesions, particularly when cysts compress or invade basal cisterns. Neuropsychiatric symptoms (e.g., personality changes, intellectual impairment) were reported in 88% of children with detailed descriptions, suggesting a considerable effect on neurocognitive development. Among the most striking observations was the significantly higher prevalence of neuropsychiatric symptoms in patients with a bad outcome (80%) compared to those with a good outcome (36%). This suggests that cognitive impairment, behavioral changes, or reduced school performance may be indicators of more severe disease progression or extensive cerebral involvement. Similarly, visual disturbances were significantly more common in the bad outcome group (94% vs. 75%), as was optic atrophy (75% vs. 36%), reinforcing the hypothesis that disease progression affecting visual pathways may be linked to worse prognosis. Additionally, ataxia (85%), cerebellar signs (57%), and pyramidal signs (92%) highlight the variable locus of disease, while rare presentations—like hemichorea (30%)—point to lesion involvement in deep nuclear structures. One of the most unexpected findings in our analysis was the complete absence of cerebellar signs in the bad outcome group, whereas 53% of patients in the good outcome group exhibited these signs. While initially counterintuitive, this observation may suggest that patients presenting with cerebellar involvement are diagnosed earlier, leading to timelier intervention and better outcomes. Alternatively, cerebellar involvement may reflect a different pathophysiological process that does not necessarily predict a severe disease course. This finding merits further investigation in future studies. Interestingly, pyramidal signs were more frequent in the bad outcome group (88% vs. 69%), suggesting that motor pathway involvement could be a marker of disease severity. Meanwhile, weakness of the lower limbs was observed in 79% of patients with a good outcome and 67% of those with a bad outcome, which may indicate that motor dysfunction alone does not necessarily predict a worse prognosis. In contrast, some symptoms appeared equally distributed between the two groups, suggesting that they are common manifestations of the disease rather than outcome-determining factors. For example, seizures were present in 75% of both groups, indicating that epileptic activity is a frequent but non-specific feature of cerebral echinococcosis. Similarly, headache (96% vs. 94%), papilledema (90% vs. 87%), and hemiparesis (90% vs. 87%) were prevalent in both groups, underscoring their role as hallmark symptoms of the disease rather than prognostic indicators. Intracranial cysts were found in 98% of cases, with the parietal region commonly involved (36%), followed by frontal (23%) and temporal (23%). Spinal hydatid cysts were found in only six cases (1,2%), while only one patient presented both spinal and intracranial cysts (0,2%). Dorsal lesions were noted in 4 patients (0,8%), lumbar involvement accounted for 2 cases (0.4%), while cervical localizations were not reported in this dataset (0%). Clinical manifestations of spinal involvement included lower limb weakness, gait disturbances, increased tendon reflexes, and in some cases, perineal reflex alterations, with loss of anal and abdominal reflexes below the umbilicus [64]. In the end, according to our findings, a higher number of localizations is significantly associated with poorer prognosis in pediatric cerebral echinococcosis: the association between cyst extension (classified as single, two, or three localizations) and clinical outcomes (good vs. bad outcome) demonstrated a direct proportionality between greater cyst extension and worsening clinical outcome (*p* < 0.001).

The overall findings of this study highlight the complexity of neurological manifestations in pediatric cerebral echinococcosis. While no single symptom emerged as a definitive predictor of outcome, the observed trends suggest that certain symptoms, particularly neuropsychiatric deficits, visual impairment, altered consciousness, and pyramidal signs, may serve as indirect markers of disease severity. These symptoms could be valuable clinical indicators for identifying patients at higher risk of poor prognosis, warranting closer monitoring and more aggressive intervention. Conversely, symptoms such as seizures, headache, papilledema, and hemiparesis appear to be general features of the disease, with limited prognostic value. The absence of cerebellar signs in patients with a bad outcome is an intriguing finding that calls for further exploration, as it may provide insights into different disease phenotypes and response to treatment. Future research should focus on developing composite prognostic models that integrate neurological symptoms, imaging findings, and laboratory biomarkers to improve risk stratification and guide therapeutic decision-making. Additionally, prospective studies are needed to validate these findings and determine whether early identification of high-risk clinical profiles could lead to improved patient outcomes.

Historically, clinicians relied on Casoni’s test and highly invasive techniques (angiography, ventriculography) which exposed children to substantial complications, including accidental rupture, meningitis, and anaphylaxis. Cerebral angiography was a radiological technique that involved injecting contrast material into the cerebral blood vessels, allowing visualization of blood flow and vascular abnormalities [102]. The most commonly used vascular access was the femoral artery. However, in diagnosing cerebral echinococcosis, it did not directly identify cysts but could reveal indirect signs such as displacement or compression of cerebral vessels due to the cystic mass [27,33,40,49]. In some cases, it could highlight an avascular area corresponding to the cyst, but it did not reveal its nature [34]. Therefore, the use of angiography lacked high specificity even if it subjected children to an invasive and risky procedure; in fact, our results demonstrated that 57% of patients diagnosed via angiography had a bad outcome (*p* < 0.001). Ventriculography was a radiological examination used to study the cerebral ventricular system. It was performed by injecting air or a radiopaque contrast agent into the cerebral ventricles and evaluating its distribution through X-rays [34,102]. This technique was used to study cerebrospinal fluid (CSF) flow, which could be altered by the presence of cysts, tumors, etc. [63]. It could show displacement or compression of the cerebral ventricles, suggesting the presence of a mass [27,45]. However, it is now an obsolete diagnostic technique no longer in use due to its highly invasive nature. According to our results, in fact, 62% of patients who underwent ventriculography experienced a bad outcome confirming its association with a higher risk of poor prognosis. Particularly, patients often died as a result of this procedure due to accidental cyst rupture, leading to severe brain infections [65]. Casoni’s test was a skin hypersensitivity reaction historically used for the diagnosis of echinococcosis [65]. It involved intradermal injection of an antigenic extract derived from Echinococcus cysts [69]. If the patient had been exposed to the parasite, a local reaction (redness and swelling) would appear within 30 min [46]. This test fell out of use due to its low specificity and sensitivity, with many false negatives; in fact, in our cohort it was described only in a few ancient cases. In contrast, computed tomography (CT) and especially magnetic resonance imaging (MRI) now enable precise, noninvasive visualization of cyst morphology, size, and relationship to critical brain structures [80]. On CT, the cyst usually appears well defined, with clear margins, a spherical shape, hypodense characteristics, and no enhancement after contrast infusion [82,88,95]. In our review, we also identified cases where the cyst exhibited a hyperdense wall on CT, typically due to calcifications [29,110]. However, in one case, the cyst had a hyperdense, ring-shaped wall, not because it was calcified, but because it had ruptured spontaneously [107]. Another unusual CT feature of the cyst is the “water lily sign” [14,97] which results from a collapsed cyst wall. When diagnosis was performed through computed tomography (CT), it was associated with a good outcome in 67% of cases (*p* < 0.007), indicating a strong correlation with a better clinical prognosis. According to our results, those patients diagnosed via MRI had notably better outcomes (93% achieved a favorable prognosis), likely reflecting timely, targeted intervention facilitated by high-resolution imaging. MRI demonstrated superiority to CT as it provides more detailed information about soft tissues and defines the anatomical position of the lesion in relation to sulci and ventricles, aiding in surgical planning. On MRI images, the cyst appears with an intensity similar to cerebrospinal fluid: hypointense on T1 and hyperintense on T2. As demonstrated by our results, the role of histopathology has evolved significantly. Previously, when radiological techniques did not provide high-resolution, definitive diagnoses, the cornerstone of diagnostic certainty relied on the postoperative histological examination of the cyst. Today, however, definitive diagnosis is primarily achieved through instrumental investigations like MRI and serologic assays, with histopathology now serving mainly as a confirmatory tool [24,53,60].

Surgery remains the principal therapy for pediatric cerebral echinococcosis [20,77,84,86]. In fact, all the pediatric patients reported were surgically treated. Over time, techniques have shifted from basic aspiration of the cyst—frequently resulting in spillage and secondary complications—to more sophisticated methods (Dowling-Orlando, Arana-Inglez) that emphasize hydrodissection, thereby minimizing rupture risk. However, this technique is still used in cases where the cyst cannot be removed intact due to its location [62]. One remarkable case involved a child with a large extraparenchymal cystic mass that resulted in visible cranial asymmetry. In this instance, the surgery was highly invasive, requiring the removal of a portion of the thickened aponeurotic galea, which had been infiltrated by parasites [16]. Our review also highlighted other surgical techniques for spinal hydatid cysts, including laminectomy or laminoplasty with cyst excision, combined with spinal cord decompression to prevent permanent neurological deficits. Yet, our data showed that children presenting with multiple or multi-regional cysts are at significantly higher risk for postoperative morbidity and overall poorer outcomes (*p* < 0.001). Previously, anti-parasitic pharmacotherapy (with albendazole or mebendazole) was administered exclusively in the postoperative setting, particularly in cases of systemic, multiple, or ruptured cysts, or when recurrence was evident. In recent years, however, pharmacotherapy has increasingly been employed not only as adjuvant treatment after surgery but also as neoadjuvant therapy, administered prior to surgical intervention with the goal of preventing recurrence. According to our results, albendazole was administered in 46% of the cohort and significantly associated with improved prognosis (*p* < 0.001), likely due to better penetration of the CNS and cystic cavities compared with mebendazole. It is reasonable that the good effectiveness of albendazole is linked to its use not only postoperatively but also as a neoadjuvant therapy, sometimes combined with corticosteroids—particularly in cases of fever or systemic infection— in order to reduce parasite viability and minimize postoperative dissemination risk [16,24,30,56,77,91]. Postoperative therapy also included praziquantel in some cases, alongside antiepileptics, especially for patients with a history of seizures.

One major strength of this work lies in its focused scope: to our knowledge, it is the first review to address exclusively cerebral echinococcosis in pediatric patients, capturing diverse global experiences. Moreover, by including a range of study designs—from case reports to observational cohorts—this analysis spans historical diagnostic and surgical practices through modern imaging and pharmacological protocols, thus offering broad insights. However, the review is constrained by methodological heterogeneity in reporting symptom details, imaging findings, and follow-up intervals. The abundance of Level 4 evidence, such as case reports, limits the ability to generalize or ascertain causal inferences. Additionally, certain data (e.g., exact onset age, comprehensive imaging metrics) were often missing, and some patients underwent older or discontinued diagnostic modalities (ventriculography) that introduced bias. Finally, pediatric cerebral echinococcosis remains rare, limiting the power of any subgroup analyses related to spinal involvement or specific surgical nuances.

Given the pleomorphic clinical presentation of cerebral echinococcosis in children, future research should evolve along several key avenues. First, the establishment of prospective multicenter registries would enable standardized collection of data regarding cyst localization and dimensions, imaging characteristics, therapeutic protocols, and long-term outcomes. In parallel, refinement of surgical techniques through comparative clinical trials appears essential, particularly in the context of multifocal disease, in order to define best practices for hydrodissection, cyst aspiration, and prophylactic strategies aimed at minimizing the risk of recurrence or anaphylaxis. Another important area concerns adjunctive pharmacological interventions: further investigations are warranted to determine the optimal timing and dosing of albendazole, as well as the potential benefit of combined regimens—such as albendazole in association with praziquantel—especially in patients with partially resected cysts or concomitant hepatic and pulmonary involvement. Finally, advances in immunological and molecular research are needed to elucidate the mechanisms underlying the heterogeneous clinical spectrum, specifically the reasons why some patients develop severe neurological compromise while others present with isolated lesions and comparatively mild symptomatology.

## 5. Conclusions

To date, this is the first systematic review and meta-analysis focusing on cerebral Echinococcosis in children. This study clarified the heterogeneous clinical course of pediatric cerebral echinococcosis, which includes prominent burdens of intracranial hypertension, seizures, focal deficits, and neuropsychiatric disturbances. Modern imaging and evolving surgical protocols have reduced mortality to below 10% in our aggregated dataset, and adjuvant albendazole therapy has emerged as a pivotal adjunct for preventing recurrence. Nonetheless, the findings emphasize the necessity for further prospective investigations to refine early detection methods, optimize surgical strategies, and tailor medical therapy—ultimately aiming to improve functional outcomes for children affected by this rare yet significant parasitic infection.

## Figures and Tables

**Table 1:** Neurological symptoms in children with echinococcosis.

Neurological Sign/Symptom	Present *n* (%)	Absent *n* (%)	Total Cases Described
Headache	323 (97%)	9 (3%)	332
Raised intracranial pressure	205 (96%)	8 (4%)	213
Vomiting	242 (96%)	10 (4%)	252
Papilledema	191 (95%)	10 (5%)	201
Hemiparesis	141 (94%)	9 (6%)	150
Weakness of lower limbs	36 (88%)	5 (12%)	41
Seizures	92 (91%)	9 (9%)	101
Aphasia	7 (50%)	7 (50%)	14
Ataxia	41 (85%)	7 (15%)	48
Cerebellar signs	13 (57%)	10 (43%)	23
Pyramidal signs	120 (92%)	10 (8%)	130
Ptosis	6 (43%)	8 (57%)	14
Optic atrophy	38 (83%)	8 (17%)	46
Cranial nerve palsies	31 (79%)	8 (21%)	39
Neuropsychiatric symptoms	61 (88%)	8 (12%)	69
Visual problems	107 (91%)	10 (9%)	117
Altered consciousness	52 (69%)	23 (31%)	75
Coma	7 (100%)	—	7
Hemichorea	3 (30%)	7 (70%)	10
Cranial asymmetry	42 (84%)	8 (16%)	50

## Data Availability

The original contributions presented in this study are included in the article/Supplementary Material. Further inquiries can be directed to the corresponding authors.

## Supplementary Materials

The following supporting information can be downloaded at https://www.mdpi.com/article/10.3390/pathogens14111144/s1, Table S1: PRISMA checklist.

## Abbreviations

The following abbreviations are used in this manuscript:

CNS	Central nervous system
CSF	Cerebrospinal fluid
CT	Computed tomography
EEG	Electroencephalography
GCS	Glasgow Coma Scale
HIV	Human immunodeficiency virus
IQR	Interquartile range
MRI	Magnetic resonance imaging
*p*	*p*-value
PRISMA	Preferred Reporting Items for Systematic Reviews and Meta-Analyses
PROSPERO	International Prospective Register of Systematic Reviews
SD	Standard deviation
